# Extensive Organ Necrosis After Thoracic Endovascular Aortic Repair for Thoracic Aortic Aneurysm: A Report of the Usefulness of Laparoscopic Indocyanine Green Intraoperative Blood Flow Assessment

**DOI:** 10.7759/cureus.22184

**Published:** 2022-02-13

**Authors:** Shoryu Takayama, Ken Ishikawa, Hisanori Kani, Satoru Takayama, Masaki Sakamoto

**Affiliations:** 1 Surgery, Nagoya Tokushukai General Hospital, Kasugai, JPN; 2 Digestive Surgery, Nagoya Tokushukai General Hospital, Kasugai, JPN; 3 Respiratory Surgery, Nagoya Tokushukai General Hospital, Kasugai, JPN; 4 General Surgery, Nagoya Tokushukai General Hospital, Kasugai, JPN

**Keywords:** tevar, celiac artery coiling, laparoscopy, icg, taa

## Abstract

An 85-year-old man underwent thoracic endovascular aortic repair (TEVAR) for a thoracic aortic aneurysm (TAA). The day after TEVAR, the patient complained of abdominal pain. Blood tests showed lactic acidosis. Contrast-enhanced CT of the abdomen showed emphysema and poor contrast areas in the lower esophagus, total stomach, and duodenum. The left lobe of the liver also showed a poorly contrasted area. Indocyanine green (ICG) intraoperative blood flow evaluation was performed by laparoscopy to evaluate how organ ischemia is and whether resection of necrotic organs is possible. It was judged that resection of the poor perfusion area would not improve prognosis because of the extensive area of poor perfusion in the ICG intraoperative perfusion evaluation. In TEVAR for TAA, embolization of the celiac artery (CA) can be performed if collateral blood flow is demonstrated. However, in this case, extensive organ necrosis happened. We discuss the cause of this case and the usefulness of ICG intraoperative blood flow assessment when ischemia is suspected.

## Introduction

If the celiac artery (CA) overlaps the stented area while performing thoracic endovascular aortic repair (TEVAR) for thoracic aortic aneurysm (TAA), coil embolization of the CA may be performed to prevent endoleak. It has been reported that embolization of the CA is a relatively safe procedure because the CA has collateral arteries with a superior mesenteric artery (SMA) [1-2]. On the other hand, complications of CA embolization are reported [3]. Necrosis of the upper GI tract, including the stomach, has a very high mortality rate and is often difficult to save even with surgical treatment [4-5]. We experienced a case of ischemic necrosis of the lower esophagus, the entire stomach, duodenum, and left lobe of the liver after CA embolization for TEVAR. There are no reports of such extensive organ necrosis associated with TEVAR. Indocyanine green (ICG) intraoperative blood flow assessment was used to evaluate the indication for treatment in this very rare case. It has been reported that appropriate bowel resection can be performed by evaluating intestinal ischemia with ICG [6]. In a rare case of extensive necrosis of upper abdominal organs, we determined the appropriate indication for surgery by ICG intraoperative blood flow evaluation.

## Case presentation

**Figure 1:**
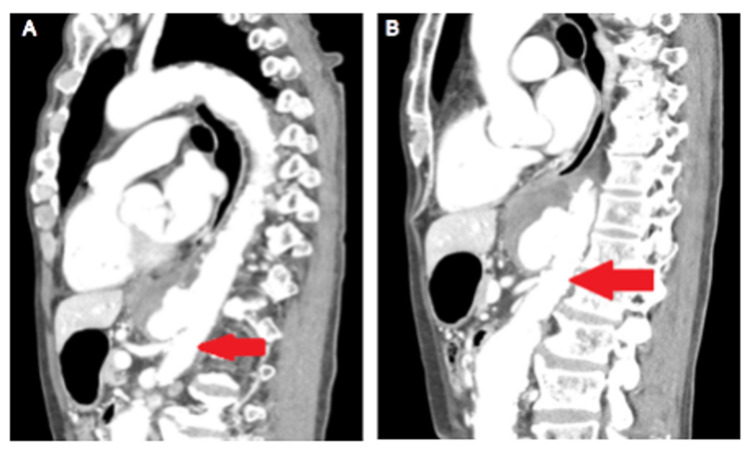
Thoracic endovascular aortic repair (TEVAR) for thoracic aortic aneurysm (TAA). (A) The patient's celiac artery (arrow) was located at the site of the stent placement. The celiac artery was embolized because it was a potential source of endoleak. (B) Embolization of the celiac artery provided a margin for stenting to the superior mesenteric artery (arrow).

An 85-year-old man with a history of myocardial infarction and dyslipidemia came to our hospital with a chief complaint of abdominal pain. A contrast-enhanced CT scan of the chest and abdomen revealed a TAA (Figure 1).

The pseudoaneurysm had already ruptured and needed to be treated immediately. Because of his advanced age, minimally invasive vascular treatment (IVR) was chosen. Although the patient had a shaggy aorta and the possibility of embolism associated with the procedure was considered high, TEVAR was scheduled after the patient was fully informed of the risks. The CA was coil embolized to prevent a type II endoleak from the CA. At that time, the SMA was contrast-enhanced to confirm the presence of collateral blood pathways, and the CA was embolized. TEVAR was completed successfully. One day after surgery, the patient complained of abdominal pain. Recurrent pain was noted in the upper abdomen. Laboratory studies are shown in Table 1.

**Table 1:** Laboratory results when the patient complained of abdominal pain. This is the result of a blood sample taken the day after embolization of the celiac artery (CA) for TEVAR. Pancreatic enzymes (Amy) and liver enzymes (AST, ALT) were elevated. In addition, lactic acid was also elevated. The results strongly suggested impaired blood flow in the CA region. APTT: Activated partial thromboplastin time; PT-INR: Prothrombin time and International Normalised Ratio.

Blood Tests	Test Results
WBC	86×102 /μl
Hemoglobin (Hb)	12.6 g/dL
Platelet (Plt)	6.8×104 /μ
Creatine Kinase (CK)	298 U/L
Aspartate Aminotransferase (AST)	738 U/L
Alanine Aminotransferase (ALT)	565 U/L
Amylase (Amy)	603 U/L
Total Protein (TP)	5.9 g/dL
Albumin (Alb)	3.4 g/dL
Creatinine (Cre)	1.03 mg/dL
Blood Urea Nitrogen (BUN)	26.6 mg/dL
Na	133 mEq/L
K	4.0 mEq/L
Cl	100 mEq/L
Blood Sugar (BS)	139 mg/dL
C-Reactive Protein (CRP)	9.02 mg/dL
PT-INR	1.44
APTT	34.2 sec
Agas	pH 7.29, Lac 14.1 mmol/L, BE -14.0 mol/L

**Figure 2:**
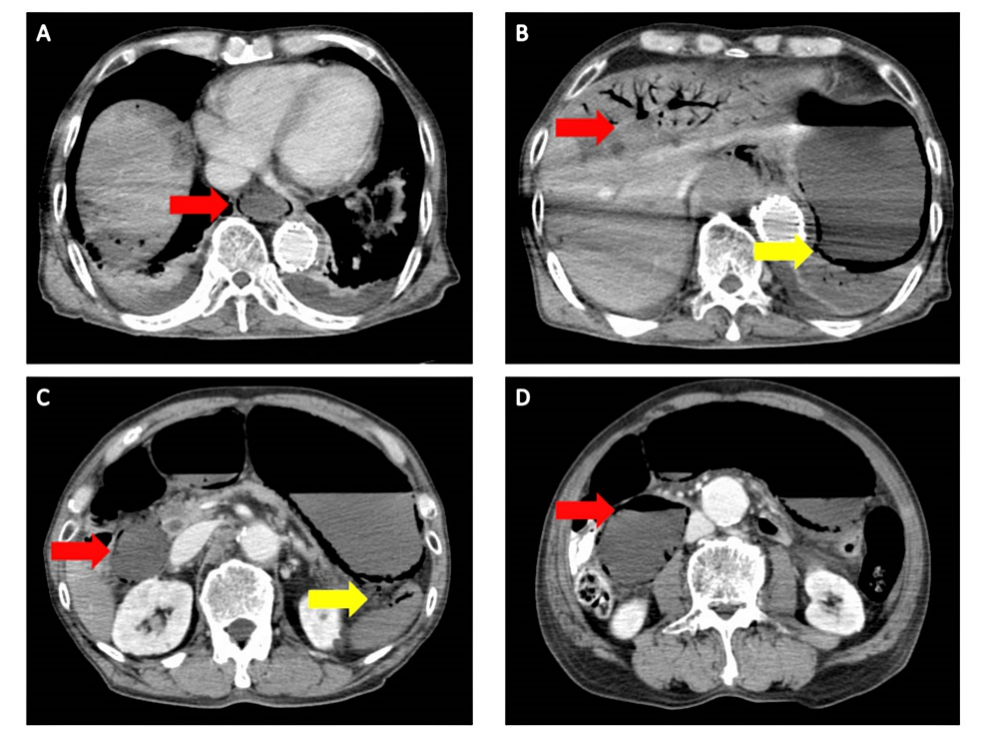
A thoracoabdominal contrast-enhanced CT after TEVAR. There was intestinal emphysema and poor contrast from the lower esophagus to the duodenum (A-D). The lateral hepatic area showed poor contrast and portal vein gas (B). The spleen also showed poor contrast and intravascular emphysema (C). TEVAR: Thoracic endovascular aortic repair.

A thoracoabdominal contrast-enhanced CT was performed, suspecting circulatory failure. There was intestinal mural emphysema and poor contrast from the lower esophagus to the duodenum. The lateral hepatic area showed poor contrast and portal vein gas. The spleen also showed poor contrast and intravascular emphysema (Figure 2).

**Figure 3:**
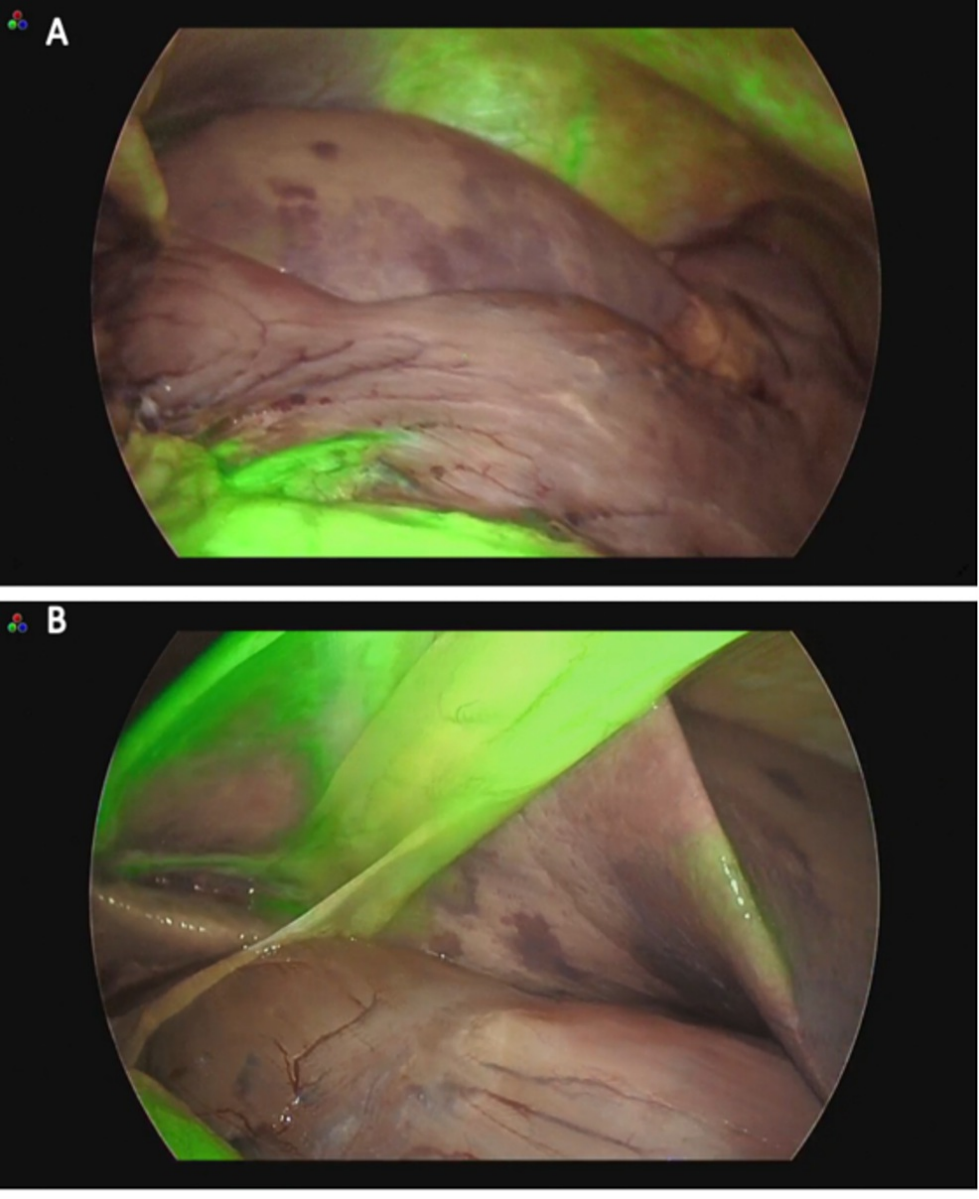
Indocyanine green (ICG) intraoperative blood flow assessment. (A) Review laparoscopy and ICG intraoperative blood flow assessment were performed to evaluate the blood flow. The area with poor perfusion on preoperative CT was not stained by ICG intraoperative perfusion evaluation, and it was judged to be completely ischemic because ICG did not stain the whole stomach at all even after 5 minutes. (B) The left lobe of the liver was not stained by ICG, bordering the hepatic condyles.

As an area of poor contrast, impairment of blood flow to organs nourished by the CA was suspected. The cardiovascular surgeon requested the GI surgeon to remove and reconstruct the necrotic organ in order to improve the lactic acidosis. Review laparoscopy was performed and ICG intraoperative blood flow assessment was performed to evaluate the blood flow. The area with poor perfusion on preoperative CT was not stained by ICG intraoperative perfusion evaluation, and it was judged to be completely ischemic because ICG did not stain the whole stomach at all even after 5 minutes. The ICG intraoperative blood flow evaluation showed that the blood flow in the extrahepatic area was also disrupted (Figure 3).

It was judged that surgery would be difficult to save the patient's life, and the surgery was terminated with observation only. Dialysis was then performed to improve the acidosis, but the patient died three days after TEVAR. The patient's family refused autopsy. The cause of the patient's death was considered to be a fatal arrhythmia due to progressive acidosis.

## Discussion

TEVAR has been found to be effective as a treatment method for thoracic descending aortic lesions (thoracic descending aortic aneurysm, penetrating atherosclerotic ulcer (PAU), blunt traumatic aortic injury (BTAI), type B dissection) and has become one of the standard treatments and surgical treatments. In particular, TEVAR is recommended preferentially over surgery for descending aortic aneurysms (including ruptures) that fulfill the anatomic requirements for TEVAR [7-8]. Treatment is predicated on the presence of a healthy, straight aortic wall of at least 20 mm on the central and peripheral sides of the lesion. In the case of TAA, the CA is sometimes embolized to obtain a peripheral site. Collateral blood flow from the SMA needs to be checked. It should be considered whether simple closure is possible or whether revascularization by bypass is necessary. In this case, collateral blood flow from the SMA was confirmed at the time of TEVAR. One day after the surgery, enhanced CT was performed, and the blood flow from the collateral circulation was blocked by some inducement. Since a shaggy aorta was noted in the preoperative contrast-enhanced CT, we considered the possibility that an atherosclerotic thrombus may have strayed into the SMA and blocked the collateral circulation after stent insertion. In this case, the patient had lactic acidosis due to ischemia-induced organ necrosis. A blood flow assessment was performed by review laparoscopy to determine if resection of the necrotic organ was feasible to improve acidosis. It has been reported that when the blood flow drops to about 30% by ICG intraoperative blood flow evaluation, the tensile strength significantly decreases, and suture failure may occur [9]. However, in this case, the blood flow was confirmed to be 0% by ICG intraoperative blood flow evaluation, and anastomosis was considered impossible. Liver and pancreatic enzymes were elevated, suggesting that the liver and pancreas were also necrotic due to impaired blood flow. If the necrotic organs were to be resected, it was necessary to resect the lower esophagus, stomach, horizontal leg of the duodenum, pancreas, spleen, and lateral hepatic segment. The quality of life (QOL) of the patient would be significantly decreased if the necrotic organs were removed, even if he could be saved. We thought it would be more effective to improve the collateral blood flow pathway by thrombolysis or other methods to prevent organ perfusion in the CA region. It has been reported that patients who underwent total gastrectomy for gastric necrosis did not survive [4], and it was appropriate not to resect necrotic tissue in the postoperative review. It has been reported that appropriate intestinal resection can be performed by evaluating intestinal ischemia with ICG and considering the surgical technique [6]. In this case, ICG intraoperative blood flow evaluation showed that blood flow from the collateral circulation had completely disappeared after TEVAR, so we were able to properly evaluate that there was no indication for surgery. Thrombus embolization of the collateral blood pathway was caused by the shaggy aorta.

## Conclusions

We experienced a case of death due to ischemia of the upper abdominal organs after TEVAR with embolization of the CA for TAA. The patient had a shaggy aorta, which was thought to be due to thrombus embolization of the collateral circulation from the SMA. We considered resection of the necrotic organ to improve acidosis. However, ICG intraoperative evaluation of blood flow showed that reconstruction was not possible, so meaningless surgery was avoided. Unfortunately, the patient died. Hence, the TEVAR treatment strategy for TAA should continue to be carefully evaluated.
